# Investigating the use of comprehensive motion monitoring for intrafraction 3D drift assessment of hypofractionated prostate cancer patients on a 1.5T magnetic resonance imaging radiotherapy system

**DOI:** 10.1016/j.phro.2024.100596

**Published:** 2024-06-06

**Authors:** Georgios Tsekas, Cornel Zachiu, Gijsbert H. Bol, Madelon van den Dobbelsteen, Lieke T.C. Meijers, Astrid L.H.M.W. van Lier, Johannes C.J. de Boer, Bas W. Raaymakers

**Affiliations:** Department of Radiotherapy, University Medical Center Utrecht, Heidelberglaan 100, 3584 CX Utrecht, The Netherlands

**Keywords:** MR-guided radiotherapy, Motion management, Intrafraction drift correction, Intrafraction motion

## Abstract

This work investigates the use of a multi-2D cine magnetic resonance imaging-based comprehensive motion monitoring (CMM) system for the assessment of prostate intrafraction 3D drifts. The data of six healthy volunteers were analyzed and the values of a clinically-relevant registration quality factor metric exported by CMM were presented. Additionally, the CMM-derived prostate motion was compared to a 3D-based reference and the 2D-3D tracking agreement was reported. Due to the low quality of SI motion tracking (often >2 mm tracking mismatch between anatomical planes) we conclude that further improvements are desirable prior to clinical introduction of CMM for prostate drift corrections.

## 1 Introduction

Prostate radiotherapy is challenged by the presence of inter- and intrafraction anatomical changes that can lead to significant treatment uncertainties. The intrafraction motion pattern can vary from progressive drifts to sudden motion events that are mainly observed in the Anterior-Posterior and Superior-Inferior directions [1]. In addition, gas pockets that lead to rectal deformations can significantly impact prostate intrafraction motion and are highly unpredictable [2], [3], [4].

Currently, scientific and clinical interest is shifting towards hypofractionated treatment schemes for prostate cancer patients, as they have reported superior oncological outcomes [5] and also require less hospital visits for patients [6]. Recently, an adaptive sub-fractionation workflow for prostate cancer patients was clinically introduced on the 1.5T MR-linac that allows for delivery of the daily fraction in two parts (sub-fractions) in the same radiotherapy treatment session [2]. Parallel to the radiation delivery MR imaging is being performed that is subsequently used for a single rigid online adaptation (after the first sub-fraction). This procedure allowed for reduction of planning margins to 2 mm [2]. Nonetheless, this adaptive scheme might not be optimal while some patients might need no adaptations and others might require more than one plan adaptation steps depending on the extent of anatomical or physiological motion.

Recently, a comprehensive motion monitoring (CMM) software package was clinically introduced on the 1.5T MR-linac [7], [8]. CMM enables motion mitigation via gating, for instance to mitigate breathing-related motion, but additionally it allows intrafraction plan adaptations by means of drift corrections in case the breathing pattern changes or the patient relaxes. Applying a drift correction entails stopping the radiation and rigidly shifting the remainder of the segments according to the motion derived from cine MR images before continuing the treatment, without any further plan optimization steps.

In the context of online adaptive radiotherapy of the prostate CMM can offer a promising alternative due to its ability to correct for intrafraction drifts of the prostate within less than a minute and only when needed. To that end, the goal of this work is to investigate the use of CMM for intrafraction 3D drift detection and evaluate its potential as a fast adaptation strategy for hypofractionated prostate cancer radiotherapy.

## 2 Materials and methods

### 2.1 Drift corrections using CMM

The CMM package (Elekta AB, Stockholm, Sweden) offers real-time 2D cine MRI-based tracking using coronal and sagittal planes acquired in an interleaved manner [7], [8]. The algorithm requires a series of 2D cine MRI images, a 3D pre-treatment MRI volume and the contour of the anatomical structure to be tracked.

Initially, image templates that represent the average motion pattern are created for each anatomical plane (one sagittal and one coronal). Then, the tracking of the target structure is achieved through an initial 3D-2D template matching step, between the 2D templates and the 3D pre-treatment MRI and subsequent 2D-2D registration steps of every incoming image frame to the corresponding image template [7]. Upon running the software the following information is exported: the tracked target centroid world coordinate positions, the average centroid motion and other quality factor metrics, such as jitter. Further details on the calculation of the jitter metric are provided in Section 2.4 of this manuscript.

Drift corrections can be applied when the centroid of the target structure drifts away from its initial position for more than a predefined distance threshold. In a clinical workflow the decision for a drift correction is based on the 3D displacement vector of the target structure. Due to the fact that SI motion is measured in both the sagittal and coronal planes, SI _sag_ and SI _cor_ respectively, CMM drift corrections rely on the averaged SI motion of the two tracked motion components.

### 2.2 2D cine MRI sequence

The default pelvic MRI scanning protocol provided by the CMM package was used for our analysis: A balanced turbo-field spin echo (bTFE) MRI sequence with a temporal resolution of five frames per second (coronal-sagittal-coronal-sagittal-coronal). Other MRI parameters are TE = 1.71 ms, TR = 3.4 ms, 40 degrees flip angle, 3 mm acquisition pixel size, 1.3 mm reconstructed pixel size and 5 mm slice thickness.

### 2.3 Volunteer data

We conducted a volunteer study using a fast MRI acquisition workflow aiming to capture common prostate motion using alternating 2D cine MRI and 3D MRI scans to cross-check the 2D-3D tracking agreement. A medical ethics approval was granted for acquiring, processing and publishing the volunteer data and results. An overview of the scanning workflow is presented in the Supplementary Material (Figure S1).

The core components of the workflow were the CMM-default bTFE 2D cine MRI sequence and a research bTFE 3D MRI sequence with prior successful applications in intrafraction 3D prostate motion tracking [3]. The bTFE 3D MRI sequence had the following parameters: TR = 4.7 ms, TE = 2.3 ms, 50 degrees flip angle, acquisition voxel size of 2 × 2 × 2.2 mm^3^ and reconstructed voxel size of 1 × 1 × 2.2 mm^3^.

A total of six healthy volunteers gave informed consent and participated in our study. The average positioning, planning and scanning time was 25 min. The volunteers were asked to remain still throughout the scanning process. The duration of the scans was 20 s (2 dynamics) for the bTFE 3D and 30 s (150 dynamics) for the 2D cine bTFE.

### 2.4 Jitter

CMM exports quality factor metrics that can characterize the tracking quality of a session, as mentioned in Section 2.1. Jitter is an important quality metric for assessing the tracking consistency in the SI motion which is measured in both sagittal and coronal planes. It is defined as the absolute value of the positional differences in the SI direction between sagittal and coronal frames, averaged over five adjacent frames for filtering out outliers. Although this metric is allowed to reach up to 5 mm before an image frame is categorized as ’failure’ and the radiation beam is turned off, large jitter values can be problematic in detecting prostate drifts, as they introduce uncertainty to the tracking and decision making.

We reported the average, interquartile range (IQR), 5th- and 95th percentile jitter values for each volunteer, as exported from the CMM software, and analyzed the temporal evolution of the jitter metric.

### 2.5 2D-3D agreement

In order to assess the 2D-3D tracking agreement the following motion analysis steps were performed per volunteer: The initial 3D MRI scan was considered as the daily scan and therefore all subsequent 3D scans were rigidly registered to the daily MRI using elastix [9], [10]. The rigid registrations were performed using prostate mask inputs, similar to the CMM, delineated on the initial 3D scan. The prostate masks were further expanded by 0.8 cm, 0.8 cm, 1.2 cm in the Anterior-Posterior (AP), Left–Right (LR), Superior-Inferior (SI) directions, similarly to the registration settings of the initial 3D-2D matching step of the CMM software. The results of the 3D image registrations can be interpreted as a set of 3D reference points of the prostate position over time [3].

**Fig. 1:**
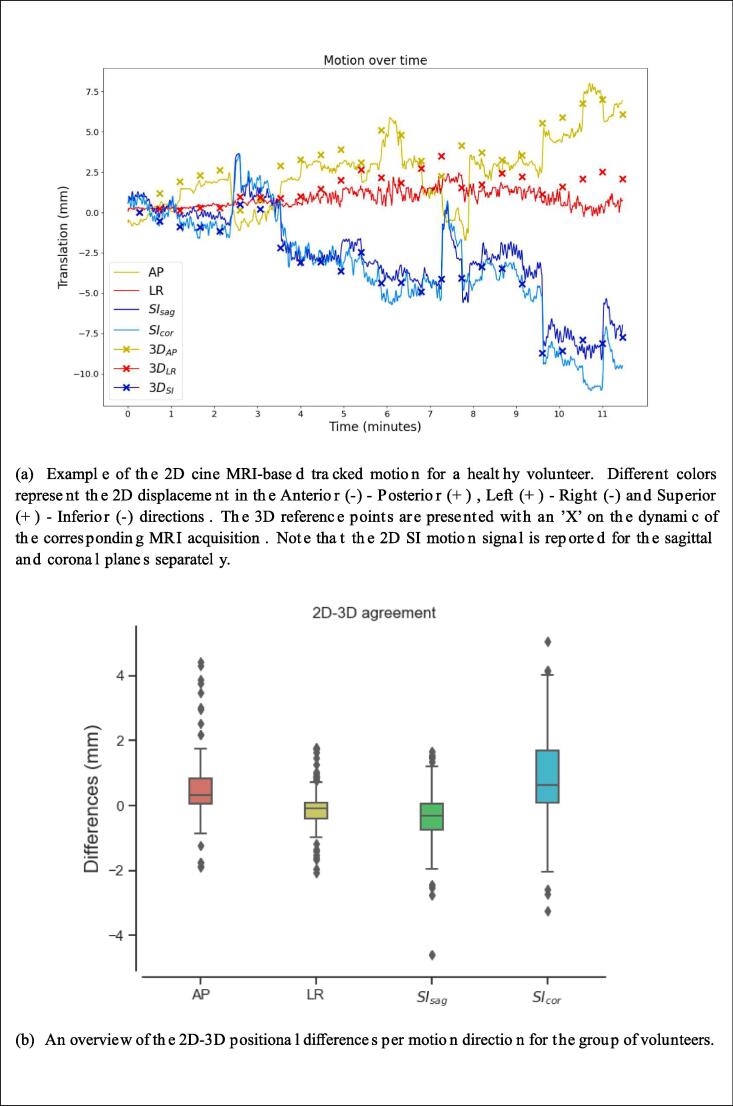
(a) Example of the 2D cine MRI-based tracked motion for a healthy volunteer. Different colors represent the 2D displacement in the Anterior (−) - Posterior (+) , Left (+) - Right (−) and Superior (+) - Inferior (−) directions. The 3D reference points are presented with an ‘X’ on the dynamic of the corresponding MRI acquisition. Note that the 2D SI motion signal is reported for the sagittal.

Then, all 2D cine MRI scans acquired over time were temporally stitched, resulting in a single 2D cine MRI signal that included motion over the total scanning time. Finally, a CMM run was performed using the initial 3D scan as the pre-treatment scan. The results of the tracking per motion direction for a volunteer alongside with the corresponding 3D reference points are depicted in Fig. 1a.

The 2D-3D tracking agreement was calculated by reporting the positional differences between the 2D- and 3D-based prostate centroid positions at each 3D reference point. In particular, every 3D reference point was compared to the last 2D-based measurements before and the first 2D-based measurements after the 3D acquisition. Prior to calculating the mismatch, the 2D cine MRI signals were smoothed using a moving average of 1 s to account for motion artifacts due to high frequency imaging. The average 2D-3D agreement was reported per motion direction for the group of volunteers.

## 3 Results

An average jitter of 1.32 mm ± 1.0 mm (mean ± std) was reported for the group of volunteers. Two out of six cases showed average jitter above 2 mm, which indicates large mismatch between SI_sag_ and SI_cor_ motion traces. Table 1 presents the statistics of the reported jitter values for each volunteer. A volunteer case with large jitter between sagittal and coronal planes is presented in the Supplementary Material (Figure S2).

**Table 1:** Jitter per volunteer: The mean, IQR, 5th- and 95th percentile values are presented per volunteer.

Volunteers	Mean ± std	IQR	5th percentile	95th percentile
Vol1	2.2 mm ± 0.9 mm	1.5 mm	0.5 mm	3.4 mm
Vol2	0.6 mm ± 0.4 mm	0.7 mm	0.1 mm	1.2 mm
Vol3	0.6 mm ± 0.4 mm	0.6 mm	0.1 mm	1.2 mm
Vol4	2.3 mm ± 1.0 mm	1.3 mm	0.5 mm	3.7 mm
Vol5	0.9 mm ± 0.6 mm	0.9 mm	0.1 mm	2.2 mm
Vol6	1.3 mm ± 0.9 mm	1.3 mm	0.2 mm	3.0 mm

Our analysis further reported average 2D-3D agreements of 0.45 mm ± 0.8 mm for the AP, −0.12 mm ± 0.49 mm for the LR, −0.36 mm ± 0.73 mm for the SI_sag_ and 0.84 mm ± 1.23 mm for the SI_cor_ motion directions respectively. The range of positional differences for each anatomical direction for all volunteers is presented in the Fig. 1b.

While the majority of the average differences in the AP, LR and SI_sag_ directions were within subvoxel ranges (<1.3 mm), larger differences between the 2D and 3D signal were observed in the SI_cor_ direction. Individual outliers can be explained by motion that may have occurred between alternating 2D and 3D MRI scan acquisitions, however larger systematic errors were reported for some volunteers between the 2D- and 3D-based prostate positions in the SI_cor_ plane.

## 4 Discussion

In this work we assessed the use of CMM for the detection of intrafraction 3D drifts of the prostate. The clinically relevant CMM-reported jitter quality factor was used for assessing the tracking quality. The large jitter present in the SI direction combined with the poor prostate visibility in the coronal plane can pose a problem for accurate prostate tracking and drift detection. Two of the volunteers reported large jitter (*>*2 mm on average), one of which reported consistent jitter of *>*1 mm for a long period of time. Large jitter values make the decision making in a clinical setup challenging, particularly when small margins are applied, although a margin analysis was not included in this work. Therefore, we believe that the current version of CMM-based prostate tracking needs further improvements before being introduced in a clinical radiotherapy workflow for intrafraction prostate drift corrections.

A fast bTFE 3D MRI acquisition protocol was also used on healthy volunteers to capture common motion patterns over time and compare the 2D cine MRI-based signal to 3D reference points. While the 3D reference scans were not used as ground truth, due to the underlying image registration uncertainties, they were used to acquire fast 3D reference points due to their prior applications in 3D prostate tracking [3]. The fact that the 2D/3D MRI acquisitions were not simultaneous is not expected to have a major effect on the tracking quality since we clinically aim at detecting prostate drifts and acting upon them within a timescale of seconds/minutes.

In the scope of our analysis the sagittal SI motion was more reliable, since it reported more consistent motion with the 3D bTFE MRI reference scans compared to the coronal plane and was further visually validated to be of superior tracking quality. This observation can be explained by the nature of intrafraction prostate motion that is primarily observed in the AP and SI directions and can thus result in large through-plane AP motion in the coronal plane [1]. In addition, sagittal planes are more robust to transient deformations such as gas pockets that are predominantly captured in the AP plane (Supplementary Material Figure S3). In our study we merely included data from six volunteers, but we believe that we captured diverse motion patterns of the prostate, including gas pockets and gradual bladder filling. We therefore believe that although we did not analyze a large amount of data, our results are indicative of the intrafraction prostate motion patterns of larger populations.

A re-initialization of the CMM image templates could potentially solve the large jitter in some cases, especially when the 2D acquisition plane of the coronal slices are affected by large through-plane drifts. Ideally, an additional intrafraction 3D MRI acquisition for validation purposes would be the best scenario, since it would serve as a robust measurement for the position of the prostate. Intrafraction rotations are another factor that might cause tracking inaccuracies [1]. Therefore, a future inclusion of 3D rotational information in the tracking of the prostate would increase tracking accuracy. Other motion tracking solutions have explored 2D cine MRI for real-time anatomy tracking on a 0.35T MR-linac [11] and multiple orthogonal cine MRI planes for intrafraction guidance [12].

In addition, the introduction of a different 2D cine MRI contrast could help increase the tracking quality. Therefore, we are developing a T2-weighted 2D cine MRI sequence that provides a better contrast in the pelvic region and we are working towards its clinical implementation for prostate tracking. Nonetheless, we believe that visual inspection by a trained observer for assessing the range of a prostate drifts before applying a drift correction should remain a necessary step in the clinical workflow.

Given the low quality of SI motion tracking we can conclude that additional technical improvements on the imaging side and a margin assessment are needed in order to use CMM for intrafraction prostate drift detection in a clinical setup.

## CRediT authorship contribution statement

**Georgios Tsekas:** Conceptualization, Methodology, Software, Formal analysis, Writing – original draft. **Cornel Zachiu:** Conceptualization, Methodology, Writing – review & editing. **Gijsbert H. Bol:** Conceptualization, Methodology, Writing – review & editing. **Madelon van den Dobbelsteen:** Writing – review & editing. **Lieke T.C. Meijers:** Methodology, Writing – review & editing. **Astrid L.H.M.W. van Lier:** Conceptualization, Methodology, Writing – review & editing. **Johannes C.J. de Boer:** Conceptualization, Methodology, Writing – review & editing. **Bas W. Raaymakers:** Conceptualization, Methodology, Writing – review & editing

## Declaration of Competing Interest

The authors declare that they have no known competing financial interests or personal relationships that could have appeared to influence the work reported in this paper.
