# Supplementary material for: Investigating the use of comprehensive motion monitoring for intrafraction 3D drift assessment of hypofractionated prostate cancer patients on a 1.5T magnetic resonance imaging radiotherapy system
Source: Phys Imaging Radiat Oncol. 2024 Jun 6;31:100596. doi: 10.1016/j.phro.2024.100596 (PMC11298924; doi:10.1016/j.phro.2024.100596)
Supplement: Supplementary data 1 [file mmc1.docx]

**Supplementary Material**


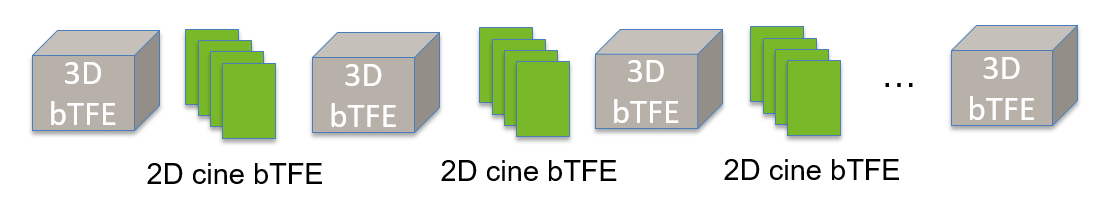


**Figure S1**: Overview of the scanning workflow of the volunteer study: A set of alternating 2D cine- and 3D MRI acquisitions were repeated, capturing prostate motion over time.


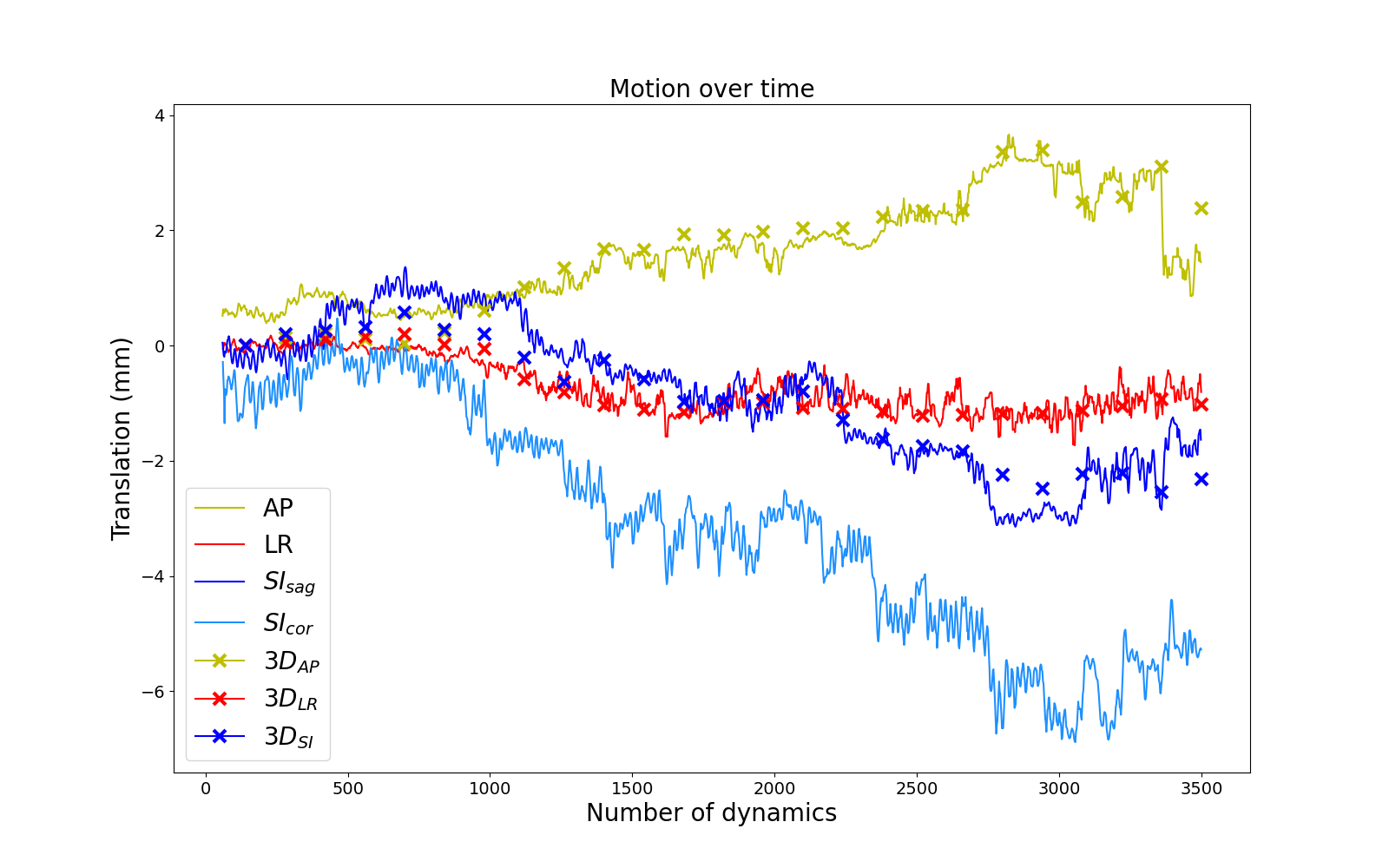


**Figure S2**: An example of a volunteer case (Vol4) where ${SI}_{sag}$and ${SI}_{cor}$ show a large mismatch. Visual inspection of the frames suggests that the coronal planes yielded inferior tracking quality, mainly due to low contrast and through-plane AP motion due to bladder filling. Different colors represent the 2D displacement in the Anterior (-) - Posterior (+) , Left (+) - Right (-) and Superior (+) - Inferior (-) directions over time. The 3D reference points are presented with an 'X' at the time of the corresponding MRI acquisition.


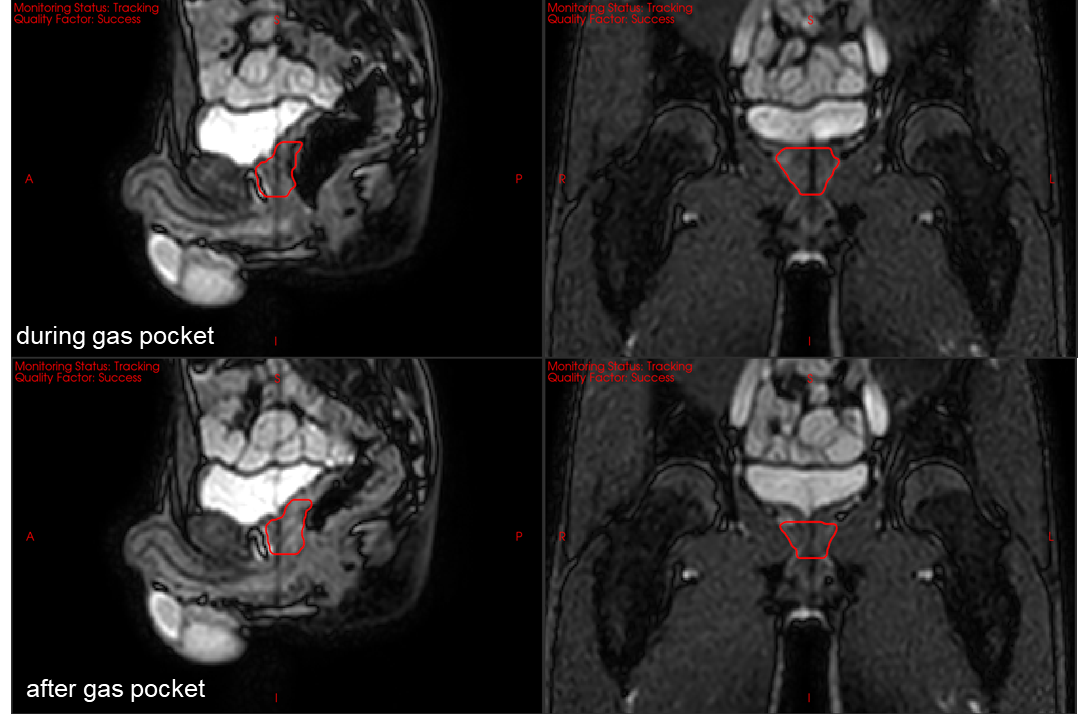


**Figure S3**: Another example of a volunteer (Vol6) reporting large jitter. A passing gas pocket caused through-plane AP motion (can be seen where the red arrows point to the extinction band on the sagittal slices) and thus affected the reported prostate centroid position on the coronal slice. On the contrary, the sagittal plane reported good tracking of the prostate and was more robust to transient motion events.
